# Proof of Concept to Clinical Confirmation: Evolving Clinical Trial Designs for Targeted Agents

**DOI:** 10.5402/2012/478607

**Published:** 2012-06-03

**Authors:** Laura Finn, Winston Tan

**Affiliations:** Hematology/Oncology, Mayo Clinic Florida, Jacksonville, FL 32224, USA

## Abstract

No single therapy benefits the majority of patients in the practice of oncology as responses differ even among patients with similar tumor types. The variety of response to therapy witnessed while treating our patients supports the concept of personalized medicine using patients' genomic and biologic information and their clinical characteristics to make informed decisions about their treatment. Personalized medicine relies on identification and confirmation of biologic targets and development of agents to target them. These targeted agents tend to focus on subsets of patients and provide improved clinical outcomes. The continued success of personalized medicine will depend on the expedited development of new agents from proof of concept to confirmation of clinical efficacy.

## 1. Introduction

Cancer is a major public health problem in the United States and worldwide [1]. In 2010 the United States had an estimated 1.53 million new cancer cases for all sites with nearly 570,000 cancer-related deaths [1]. As the second leading cause of death for all age groups in the United States, continued efforts to improve cancer therapy are critical to improve patient quality of life and survival outcomes.

The development of cancer therapy stems from the design and results of clinical trials. Traditional clinical trial design was primarily developed in the 1970s when few cancer therapies were available [2, 3]. This traditional design is derived from an evidence-based medicine module treating patient populations with similar tumor tissue types [2, 4]. Development of a therapeutic agent with intent to obtain approval from the Federal Drug Administration (FDA) tends to progress stepwise through preclinical testing for proof of concept and pharmacologic testing included in or followed by phase 1 testing of drug dosing and safety, phase 2 study of clinical efficacy, and phase 3 studies that traditionally demonstrate a clinical benefit compared to placebo or current standard of care [4, 5]. Historically this pattern of clinical investigation has been time consuming, taking 20 years or more to complete from development of concept to proof of clinical benefit, has been expensive, and depends on patient resources as the number of patients able and willing to participate in clinical trials, especially early when safety and pharmacokinetic trials are limited [4–8].

Overall survival (OS) is often acknowledged as the traditional endpoint for phase 3 clinical trials leading to FDA approval [9–11]. Initially, in the early 1980s approval of drugs by the FDA was based on tumor response, but the risk of toxicity from cancer therapy did not support this endpoint leading to a requirement of improvement in survival or patient symptoms [2, 10]. Between January 1, 1990, and November 1, 2002, nonsurvival endpoints were the basis of approval for 75% of oncology drug approvals including tumor response, time to progression (TTP), and relief of tumor-related symptoms [10, 12–14].

In this paper we will review how development of targeted agents has changed from the discovery of the first targeted therapy, imatinib, to the approval of new targeted therapies in prostate cancer. We will also discuss potential ways to expedite development of new therapy as the landscape of cancer treatment progresses beyond traditional cytotoxic chemotherapy to improved targeted agents that require biomarker identification and patient stratification that allows matching of the right patients to the right therapy [15–17].

## 2. The Discovery of Targeted Agents

### 2.1. Imatinib

The hematologic stem cell disorder chronic myeloid leukemia (CML) was first described in 1845 [18]. Early treatments included potassium arsenate, interferon alpha, hydroxyurea, and busulfan with modest results in the chronic phase of the disease and limited benefit in advanced disease [18, 19]. Through high-resolution karyotyping, a small deletion at the end of chromosome 22 was identified and the first direct link between a specific chromosomal abnormality and any malignancy was made with the discovery of the Philadelphia chromosome by Peter Nowell and David Hungerford in1960 [18, 20]. Ten years later, 1973, Janet Rowley discovered the reciprocal translocation t(9; 22)(q34; 11) through the technique of chromosomal banding [18, 21].

Almost another ten years passed before the BCR-ABL fusion gene was discovered in 1982 and nearly another decade later was identified as the cause of CML in mice in 1990 [18]. A 2-phenylaminopyrimidine derivative CGP53716 was found to inhibit the PDGF receptor and v-ABL in vitro and in vivo and became known as signal transduction inhibitor 571 (SDI571) as studies demonstrated its inhibition of cellular growth in CML [22–26]. In June 1998 a phase 1/2 clinical trial in chronic phase CML patients resistant to interferon therapy was initiated and along with a follow-up phase II trial showed that SDI571 was very effective in treating chronic phase CML and had palliative effects in the acute blast crisis phase [27–29]. By an accelerated approval process, SDI571 or imatinib was approved by the FDA in May 2001 which was the end of a 50-year effort from the discovery of the Philadelphia chromosome to approval of the first targeted therapy [18].

### 2.2. Trastuzumab

The first solid organ malignancy to have an FDA approved targeted therapy was breast cancer [30]. The discovery that avian erythroblastosis tumor viruses encoded the oncogene HER-1 implicated receptor tyrosine kinases in cancer development in the 1980s [31]. This was followed by the discovery of the neu gene from chemically induced rat neuron/glioblastomas [31, 32] which was found to have homology with the erbB receptor tyrosine kinases [33]. The human counterpart to the rat neu oncogene was localized to chromosome 7q21 and named HER2 in 1985 [31] and that same year was found to be overamplified in human mammary carcinoma [34].

HER2 overexpression is found on 20% of breast cancer cells, the majority due to gene amplification [35]. This discovery of HER-2 and its association with a poor prognosis prompted the investigation of this tyrosine kinase receptor for targeted therapy. Murine monoclonal antibodies targeting the extracellular domain of HER2 created by the then young pharmaceutical company Genentech Inc. inhibited the growth of cell lines that overexpressed HER2 in vitro and in vivo. To reduce the immunogenicity of this antibody, it was fused to a human IgG creating a humanized monoclonal antibody called trastuzumab [30].

The first phase 1 study of trastuzumab began in 1992 followed by additional phase 1 trials [30] and small phase 2 studies [36, 37]. Pivotal, large multinational phase 2 studies completed at the same time confirmed the efficacy and safety of trastuzumab. Enrolling 222 women between April 1995 and September 1996 with HER2 overexpressing, pretreated, metastatic breast cancer from 7 different countries, Cobleigh et al. [38] completed a phase 2 study with a primary endpoint of tumor response. Similar to the smaller, earlier studies the ORR was 15%, median duration of response was 9 months, and the median survival was 13 months [38]. The responses in these studies were significant due to enrollment of patients with poor risk factors [36–38].

The synergy of trastuzumab with cytotoxic chemotherapy agents seen in early studies [37, 39, 40] led to the pivotal multinational phase 3 study comparing chemotherapy in combination with trastuzumab to chemotherapy alone. Among 12 countries, the first patient was enrolled June 12, 1995 and accrued a total of 469 patients with HER2-positive metastatic breast cancer. Patients received an anthracycline or paclitaxel with cyclophosphamide. The primary endpoint for median TTP for chemotherapy and trastuzumab was 7.4 months compared to 4.6 months for chemotherapy alone. The addition of trastuzumab to anthracycline therapy increased ORR from 42% to 56%, while addition of trastuzumab to paclitaxel increased ORR from 17% to 41%. The OS combining both groups was 20 months for chemotherapy and 25 months for combination therapy [41]. These pivotal multinational trials were followed by a phase 2 study of trastuzumab as first-line monotherapy in HER-2-positive metastatic breast cancer. Evaluation of secondary endpoints demonstrated ORR of 26% for all patients in the study. As seen in the preceding studies, patients with 3+ immunohistochemical staining for HER-2 or FISH for gene amplification had the higher responses [42].

In September 1998 the FDA approved trastuzumab for use in women with metastatic breast cancer whose tumors overexpress the HER-2 protein. FDA-approved indications included treatment of patients as first-line therapy in combination with paclitaxel and as a single agent after prior chemotherapy. This initial approval for trastuzumab was followed in December 2000 by enrollment into phase 3 trials using trastuzumab in the adjuvant treatment of early-stage HER-2-positive breast cancer. These studies were sponsored by the National Cancer Institute and conducted by researchers of the National Surgical Adjuvant Breast and Bowel Project (NSABP B-31) and the North Central Cancer Treatment Group (NCCTG N9831) [43]. A third adjuvant trial, the Herceptin Adjuvant trial (HERA), started enrolling in Europe in March 2001 [44].

The results of all three trials were reported in the same edition of the New England Journal of Medicine in 2005. Romond et al. [43] reported the joint analysis of the results of the NSABP B-31 and NCCTG N9831 trials as their designs were similar, comparing doxorubicin and cyclophosphamide followed by paclitaxel with or without trastuzumab. The primary endpoint of these trials was disease-free survival (DFS). The DFS in the trastuzumab arm 4 years after randomization was 85.3% versus 67.1% in the control arm [43]. The HERA trial reported the interim analysis results of 1 year of trastuzumab versus observation administered after completion of all chemotherapy and radiation therapy. Over 5,000 women were enrolled to evaluate a primary endpoint of DFS which was 85.8% versus 77.4% in the control arm [44]. Based on the joint analysis of the NSABP B-31 and NCCTG N9831 trials, the FDA approved trastuzumab as part of a treatment regimen containing doxorubicin, cyclophosphamide, and paclitaxel for adjuvant treatment of patients with early-stage HER2-positive, node-positive breast cancer in November 2006. Based on the HERA interim analysis, the FDA added approval as a single agent in HER2-positive, node-positive breast cancer or node-negative breast cancer with high-risk features following chemotherapy.

The most recent approval for trastuzumab in breast cancer came in May 2008 based on the results of the Breast Cancer International Research Group 006 (BCIRG-006) trial that was published earlier in 2011 in the New England Journal of Medicine. This is the fourth large multinational trial evaluating anthracycline chemotherapy alone versus trastuzumab with anthracycline or docetaxel chemotherapy again demonstrating a DFS and OS benefit with combination therapy [45]. The discovery of the HER2 gene in 1984 to the last FDA-approved indication for trastuzumab in breast cancer in 2008 spanned almost 25 years.

Clinical trial design and momentum has become more streamlined than the cumbersome process experienced with the development of early targeted agents imatinib and trastuzumab. The development, study, and approval process of these drugs were prolonged for many reasons including dependency on newly developed technology, inexperience with the new targeted agents, patients willingness to be involved in clinical trials, and large-scale development requirements on pharmaceutical companies [2, 4–6, 17]. We are seeing a more efficient process of development of both chemotherapy agents and targeted agents, highlighted recently by the advances made in prostate cancer therapeutics.

### 2.3. Cabazitaxel

Taxanes are cytotoxic chemotherapy agents in use for at least the past 20 years for treatment of solid organ malignances as either a single agent or as part of a multiagent regimen [46]. A limitation to their use, especially with first-generation taxanes, is their binding affinity to multidrug resistance (MDR) proteins or efflux pumps [46, 47]. The taxane XRP6258 was selected for development for its low affinity binding to these MDR proteins [46, 47]. In phase 1 study of 25 pretreated patients with advanced solid tumors, there were two patients in which anticancer activity was noted, both with refractory prostate cancer [46]. Cabazitaxel was well tolerated amongst the patients treated in the phase 1 trial. The phase 2 dosing of 20 mg/m^2^ was chosen as patients had grade 4 neutropenia at the 25 mg/m^2^ dosing following the traditional 3 × 3 × 3 phase 1 clinical trial design [46]. Early recognition of response in the prostate cancer population led to a critical decision to advance to phase 3 study without a preceding phase 2 evaluation [48]. The TROPIC trial opened January 2007 and completed September 2009 enrolling 755 patients from 26 countries comparing prednisone plus cabazitaxel versus mitoxantrone for metastatic castration-resistant prostate cancer after progression following docetaxel treatment and previous hormone therapy. The primary endpoint was OS which was 15.1 months with median progression-free survival (PFS) of 2.8 months in the cabazitaxel group compared to OS of 12.7 months with PFS of 1.4 months in the mitoxantrone group [49]. The phase 1 data for cabazitaxel was presented at the American Society of Clinical Oncology conference May 2001, and 9 years later the FDA approved cabazitaxel for use in combination with prednisone for hormone refractory prostate cancer after or during docetaxel chemotherapy in June 2010.

### 2.4. Sipuleucel-T

The second recent prostate cancer therapy to receive approval by the FDA was the immunotherapy sipuleucel-T. The early promise of immunotherapy with autologous dendritic cells was demonstrated in sequential phase 1 and phase 2 studies published in December 2000 [50] after clinical studies in the late 1990s demonstrated that dendritic cell therapy could elicit a clinically beneficial immune response [51, 52]. The phase 1 and phase 2 studies by Small et al. [50] demonstrated that the treatment was well tolerated. In the phase 2 study, patients received sipuleucel-T from a leukapheresed product after phase 1 infusion of increasing doses. In this trial the TTP correlated with the development of an immune response and with the dose of dendritic cells received [50]. This preliminary evidence warranted further exploration with several phase 3 clinical trials that its May 2010 FDA approval was based upon, ten years after the phase 1 and 2 clinical studies. The first, published in 2006, enrolled 127 patients with asymptomatic metastatic hormone refractory prostate cancer and randomized them in a 2 : 1 fashion to receive sipuleucel-T or placebo with TTP as the primary endpoint. The study did not achieve statistical significance for this endpoint; however, it did suggest a survival advantage in the therapeutic group [53]. Integrated data from 2 randomized phase 3 trials with the primary endpoint of TTP and a secondary survival analysis after a 36-month followup in the intention to treat population supported this observation. The median survival was 23.2 months for sipuleucel-T and 18.9 months for placebo; however, again the studies did not meet statistical significance for the primary endpoint, demonstrating a trend to delay in disease progression [54]. Finally the Immunotherapy for Prostate Adenocarcinoma Treatment study (IMPACT) was published in 2010, enrolling 512 patients to treatment or placebo in a 2 : 1 fashion with a primary endpoint of OS. There was a statistically significant 4-month improvement in median survival for sipuleucel-T treated patients, 25.8 months versus 21.7 months. Again, no effect on TTP was observed [55].

### 2.5. Abiraterone

In 2011 the oral selective and irreversible inhibitor of CYP17, abiraterone acetate, was approved in combination with prednisone for castrate-resistant prostate cancer after treatment with docetaxel chemotherapy. After preclinical studies demonstrated evidence of 17a-hydroxylation inhibition in the late 1980s and early 1990s, abiraterone acetate was tested for the first time in a series of phase 1 clinical trials in both castrate and noncastrate males demonstrating further suppression of testosterone in castrate males and compensatory hypersecretion of luteinising hormone that overcome testosterone suppression in the noncastrate males [56]. The activity of abiraterone acetate in castrate resistant prostate cancer was again seen in phase 1/2 clinical trial design by Attard et al. [57] in chemotherapy-naïve patients where a 50% decline in PSA was seen in a majority of patients along with a decrease in measurements of circulating tumor cells and evidence of radiologic responses [57]. Additional analysis demonstrated reversal of resistance in 33% of patients with the addition of dexamethasone at disease progression. Similar response in PSA decline was also seen in patients who previously received ketoconazole therapy [58]. Further encouraging evidence of the therapeutic efficacy of abiraterone acetate was seen in two multicenter phase 2 trials both with primary end points of 50% reduction in PSA in patients with castrate resistant prostate cancer previously treated with docetaxel [59, 60]. The randomized, placebo-controlled, multicenter, phase 3 trial that led to FDA approval enrolled 1195 patients to receive abiraterone acetate with prednisone or placebo with prednisone in a 2 : 1 ratio. Treatment was given until there was a 25% increase in PSA which defined disease progression, unacceptable toxicity, or withdrawal. The interim analysis after 552 deaths demonstrated a statistically significant improvement in OS and the updated survival analysis after 774 deaths confirmed the survival benefit. The interim analysis demonstrated a median survival of 14.8 months for abiraterone acetate versus 10.9 months for placebo; the updated analysis also demonstrated a 4-month median survival advantage of 15.8 months versus 11.2 months [61].

### 2.6. Future

The future of cancer therapy development and expedited drug approval will likely be based to a greater extent on identification of small subsets of patients to whom targeted therapy can be designed and clinical trials tailored to rapid identification of clinical benefit [15, 17]. This pathway of drug development is exemplified by the recent discovery and approval of the anaplastic lymphoma kinase (ALK) inhibitor crizotinib. The identification of the EML4-ALK fusion gene in nonsmall cell lung cancer (NSCLC) was reported in 2007 [62], and within 3 years ORR of 57% and median 6 months PFS of 72% in pretreated patients with NSCLC with the EML4-ALK translocation was reported [63]. There was also early identification of mechanisms of resistance [64]. Within 4 years, after presentation at the 2011 American Society of Clinical Oncology annual meeting of initial phase 2 study results of crizotinib in ALK-positive NSCLC, the FDA approved for treatment of locally advanced or metastatic disease in August 2011 [65]. This timeline from proof of concept to proof of clinical efficacy is in stark contrast to the decades it took to develop early targeted agents including imatinib and trastuzumab described earlier [66].

## 3. Conclusion

From inception of concept to phase 3 trial design, it took an average of 10 years for FDA approval of these new prostate cancer drugs which is an improvement in providing earlier access to innovative therapy but still indicates that there is continued room for improvement [67]. Factors that may decrease time to drug approval include improved biomarker identification to identify small subsets of target patients as was seen in the development of crizotinib. Identification of such biomarkers will allow for trials designed and directed to particular patient populations increasing the likelihood of a clinical outcome rather than phase 1 trials designed to search for target patient populations, a daunting task for the multitude of therapies in phase 1 trial design at any given time [2, 6, 17, 68, 69]. Identification of patients for enrollment into clinical trial may also improve, not only with identification of patients that harbor target biomarkers but also with early discussions with patients about willingness to enroll in clinical trials and early collection of this information [2, 68]. Clinical trial designs can adopt a more integrated model that is adaptive to available knowledge shared during the development of studies and adaptive to improved knowledge regarding genomic heterogeneity of tumors [8, 16, 17]. In addition information sharing amongst investigators can be beneficial to avoid re-creation or reporting of identical clinical trials allowing for earlier trial design advancement leading to an expedited pathway of therapy discovery to validation [4, 68].
